# Modulation of Bacterial sRNAs Activity by Epigenetic Modifications: Inputs from the Eukaryotic miRNAs

**DOI:** 10.3390/genes10010022

**Published:** 2018-12-31

**Authors:** Brice Felden, David Gilot

**Affiliations:** 1 University of Rennes 1, Inserm, BRM (Bacterial Regulatory RNAs and Medicine), UMR_S 1230, F-35043 Rennes, France; 2 CNRS UMR 6290, IGDR, University of Rennes 1, F-35043 Rennes, France

**Keywords:** RNA modifications, epigenetics, bacterial regulatory RNAs, miRNAs, editing, methylation, biogenesis, trans-acting RNA

## Abstract

Trans-encoded bacterial regulatory RNAs (sRNAs) are functional analogues of eukaryotic microRNAs (miRNAs). These RNA classes act by base-pairing complementarity with their RNA targets to modulate gene expression (transcription, half-life and/or translation). Based on base-pairing, algorithms predict binding and the impact of small RNAs on targeted-RNAs expression and fate. However, other actors are involved such as RNA binding proteins and epigenetic modifications of the targeted and small RNAs. Post-transcriptional base modifications are widespread in all living organisms where they lower undesired RNA folds through conformation adjustments and influence RNA pairing and stability, especially if remodeling their ends. In bacteria, sRNAs possess RNA modifications either internally (methylation, pseudouridinylation) or at their ends. Nicotinamide adenine dinucleotide were detected at 5′-ends, and polyadenylation can occur at 3′-ends. Eukaryotic miRNAs possess *N*^6^-methyladenosine (m^6^A), A editing into I, and non-templated addition of uridines at their 3′-ends. Biological functions and enzymes involved in those sRNA and micro RNA epigenetic modifications, when known, are presented and challenged.

## 1. Introduction

All living organisms require regulation of gene expression to survive, grow, and spread in their dynamic and precarious environments, e.g., the survival of the fittest. A large portion of the eukaryotic genomes is expressed as regulatory RNAs [1], and bacterial/archaeal genomes produce hundreds of them [2]. The complexity of higher organisms correlates well with their extent of riboregulators rather than by the number of protein genes. For incessant and fast transcriptomic and metabolic adaptations to the cell needs set by the environment, RNA-based regulation of gene expression is widely used. It may be advantageous to use regulatory RNAs when immediate and reversible responses to external conditions are required. To make an analogy, RNA-based RNA regulations look like to the sympathetic nervous system, which assumes immediate responses to stimuli. In contrast, neo-transcription imitates the hormonal system that is time- and energy-consuming and usually involved in long-term effects.

In bacteria, regulatory RNAs (sRNAs) are also essential players in many physiological and adaptive responses and are central components of networks controlling bacterial responses to stress [3]. A large subset of riboregulators act through base pairing with target RNAs to modulate gene expression. A major paradigm for RNA-based regulation in both eukaryotes and prokaryotes are the sRNAs that pair with messenger RNAs (mRNAs), leading to changes in target mRNA stability and/or translation [4]. They can be categorized in cis-acting (antisense RNAs) and trans-acting RNAs (sRNAs). Cis-acting RNAs are transcribed from the complementary strand of their targeted gene [5] and will not be discussed here. In bacteria and archaea, the most studied riboregulators are the sRNAs. Downregulation of target RNA expression can occur when a sRNA pairs to, or close to the ribosome binding site (RBS), efficiently preventing translation initiation. This interaction often leads to cleavage of the mRNA target through endoribonucleases, to ensure irreversible regulation. Conversely, sRNA-target mRNA interactions can destabilize mRNA structures that sequester RBS, in turn enhancing translation initiation. Since the eukaryotic ribosomes do not interact, via the small-subunit ribosomal RNA (rRNA), with the mRNAs to be translated, such sRNA-mediated regulatory mechanisms are lacking for the microRNAs (miRNAs) that usually interact elsewhere onto their target mRNAs (mainly on 3′-UTRs [3′-untranslated regions]). Nonetheless, rare exceptions have been described for miRNA promoting translation. Promotion of protein synthesis can result from conformational change of the target mRNA following sRNA or miRNA pairings, but, in prokaryotes, sRNAs usually trigger translation initiation silencing.

**Figure 1:**
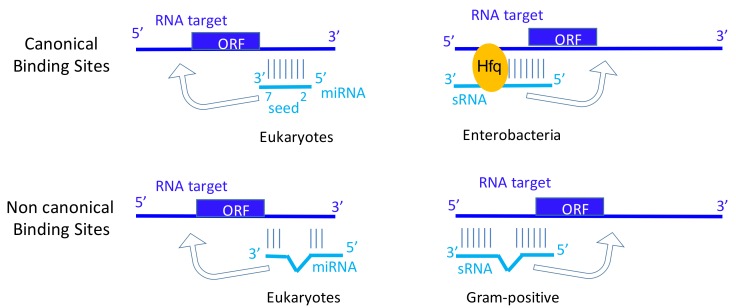
Canonical and non-canonical binding sites for micro RNA (miRNA) and regulatory RNA (sRNA). Target RNAs can be messenger RNAs (mRNAs) (as shown) or other non-coding RNAs (ncRNAs). The miRNA-Induced Silencing Complex (miRISC), containing at least Argonaute proteins, is required for base pairing between seed sequence of the miRNA and the targeted-RNA. For non-canonical binding sites, the nature of the miRISC is poorly studied. In Enterobacteria, Hfq is involved in the base pairing between sRNA and targeted-RNA, in contrast to sRNAs acting in Gram-positive bacteria that usually possess extended pairing domains. miRNAs and sRNAs bind to their target mRNAs at the 3′-UTR (3′-untranslated regions) or translation initiation sites, respectively. However, miRNAs can also bind at 5′UTRs, sRNAs at 3′UTRs, and both miRNAs and sRNAs within coding sequence regions (arrows). ORF: open reading frame.

Epitranscriptomics correspond to RNA chemical modifications and post-transcriptional nucleotide additions that are involved in gene expression regulation, including pre-mRNA splicing, degradation and translation. Post-transcriptional RNA modifications provide another layer of gene expression monitoring. More than 160 chemical modifications can be detected on RNAs [6]. They can impact RNA structure, conformation dynamics, function and interactions with their macromolecular targets, including mRNAs for the sRNAs and miRNAs. The main purpose of this review is to provide parallels and differences between the bacterial/archaeal sRNA post-transcriptional modifications, and the eukaryotic miRNA modifications. Both the bacterial/archaeal sRNAs and the miRNAs act by base pairing with target mRNAs, and both interact with their RNA targets by imperfect, interrupted pairings (Figure 1). We will not present and discuss the numerous RNA modifications in rRNA and transfer RNA (tRNA). Shared properties of trans-acting bacterial regulatory RNAs and eukaryotic miRNAs, focusing on their similarities and differences about their RNA modifications, are discussed.

## 2. Biogenesis and Functions of Bacterial Trans-Acting RNAs and Eukaryotic micro RNAs

**Figure 2:**
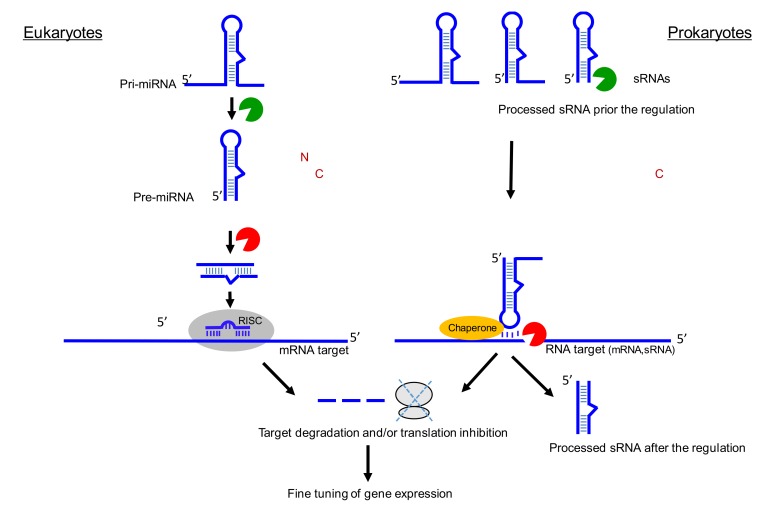
Parallels between miRNA and sRNA biogenesis and functions. For miRNA, two enzymes (Drosha and Dicer) are usually required for the biogenesis of a double strand RNA. Only one RNA strand is associated with argonaute protein. The RISC (RNA-induced silencing complex) allows the base pairing with the target (here mRNA) and the recruitment of other proteins involved in translation inhibition and mRNA decay. ‘N’ for nucleus and ‘C’ for cytosol. For sRNA, various RNases (green) can cleave triphosphorylated primary transcripts, and each matured RNA can hold their own functions in stress, metabolism, or virulence. Base-pairing with the target(s) (here mRNA) can be facilitated by dedicated chaperones (orange) than can be followed by target RNA cleavage (red) and/or by modifying translation initiation onto the target mRNA. Both miRNA and sRNA fine-tune gene expression. Note that the sRNA processing steps are not well-examined in many cases and can occur either prior and/or after binding onto their RNA targets.

Figure 2 schematically outlines similarities between miRNA and sRNA biogenesis, mechanisms, and functions in pro and eukaryotes. RNases are required for biogenesis and maturation at both instances and are required during target gene regulation. Dedicated chaperones can also be at play to assist recognition and pairing. 

### 2.1. sRNA Biogenesis

sRNA is a heterogeneous category in terms of size, ranging from ~50 to ~600 nt in lengths. sRNA biogenesis initiates with promoter-based transcription, with primary transcripts that can be processed to shorter sRNAs, by ribonuclease processing [7]. Thus, their expression patterns during bacterial growth can be complex and variable in terms of lengths and RNA forms.

### 2.2. sRNA Functions

sRNA expression usually reshapes bacterial transcriptomes and metabolisms, especially in response to specific environmental cues. sRNAs can be implicated in changes in carbon source availability, membrane stress, stationary-phase growth, bacterial virulence [8], and antibiotic resistance [9]. Single sRNAs can target multiple RNAs (both mRNAs and other sRNAs), and single mRNAs can be targeted by multiple sRNAs. They exert their regulatory functions by interacting with their mRNA or sRNA targets through binding sites extending from several to a dozen nucleotides with partial, interrupted, or complete complementarity to their targets [10]. Co-degradations between sRNAs and their RNA targets can occur, to favor timely and reversible regulations. sRNA function can rely on RNA chaperones to increase their stability and assist them during target regulation [11]. 

### 2.3. Micro RNA Genesis

MicroRNAs are short (~22 nucleotides) non-coding RNAs involved in gene expression regulation (Bartel, 2018). miRNA biogenesis involves several steps [12]. As miRNA sequences are localized in a local hairpin in a nascent transcript (primary miRNA, pri-miRNA), endonucleolytic reactions are required to generate functional miRNAs. The RNase III Drosha first cleaves the pri-miRNA to release an approximatively 70 nt hairpin-shaped RNA (pre-miRNA) [13]. The Drosha cofactor DGCR8 (also known as Pasha) promotes the generation of hairpin by Drosha. The resulting hairpin displays a 2 nt overhang at the 3′ terminus. Next, the pre-miRNA is exported to the cytoplasm by exportin 5 protein. Dicer, a cytoplasmic RNase III, converts the pre-miRNA into a small RNA duplex. To generate such miRNA duplexes, human Dicer binds the 2 nt 3′ overhang of hairpin resulting from Drosha. Then, the resulting miRNA duplex is loaded on to Argonaute. At this step, only one strand is conserved into Argonaute to form an active RNA-induced silencing complex (RISC) [14].

In recent years, next-generation sequencing analysis has revealed variability in the individual mature miRNA sequences derived from the same precursor (pre-miRNA), giving rise to the term “isomiRs” [15]. IsomiRs differ by the exact nature of their ends influencing the length. Nevertheless, isomiRs share a common core sequence (~18 nucleotides). The sequence diversification in mature miRNAs can be produced through a variety of mechanisms, including imprecise or alternative cropping of the precursor or non-canonical miRNA biogenesis. In the latter case, the canonical cascade Drosha-Exportin5-Dicer is not fully required for the miRNA biogenesis. 

### 2.4. Micro RNA Functions

MicroRNAs play critical roles in many biological processes and diseases, including oncogenesis. miRNAs guide the silencing Argonaute protein complex to messenger RNA targets to trigger post-transcriptional repression. Perfect base pairing between miRNA seed sequences (nucleotides 2–7) and miRNA response elements (MREs) of an mRNA leads to translation inhibition and mediates mRNA decay [16]. Imperfect seed pairings are also functional, although the interaction with Argonaute and the repression are weaker [17]. In addition to these ‘miRNA-mRNA’ interactions leading to post-transcriptional repression, additional studies have identified many non-canonical binding sites, referred as non-canonical MREs [18,19,20]. These MREs lead to imperfect base pairing between the seed sequence of the miRNA and the targeted-RNA. 3′-compensatory-pairing (>4–5 pairs) is described, probably to stabilize the interaction between the miRNAs and their targets [4]. We would like to propose parallels between canonical binding sites in miRNA and in sRNA from enterobacteria requiring short pairings, and between non-canonical binding sites between miRNA and sRNAs from Gram-positive bacteria that require extended and interrupted pairing interactions (Figure 1). 

Two recent meta-analyses confirmed that non-canonical MREs efficiently interact with miRNAs [16,21]. While the first study considered these non-canonical binding sites as `non-functional’ because they do not mediate mRNA decay and show no sequence conservation [16], the second study advocated for biologically relevant roles because they identified a few types of non-canonical binding site that could downregulate mRNA expression [21]. 

Considering the number of binding sites for a miRNA and the usually low level of miRNA expression level per cell, it has been proposed that RNAs sharing the same MRE, compete to bind miRNA (competing endogenous RNAs; ceRNA). In this concept, the miRNA is the limiting factor [22,23]. Thus, it is useful to quantify the expression level of miRNA of interest per cell and its eventual modifications (see part 2) affecting its activity. Due to technical difficulties to identify *in cellulo* or predict *in silico* all the mRNA binding sites for a given miRNA, it is challenging to establish the physiologic repertoire of RNAs targeted by a miRNA. To overcome these limitations, gain- and loss-of-function experiments have been performed to identify main targets for miRNA. However, if we assume that competition between RNAs to bind miRNA exists, it is important to keep in mind that gain-of-function experiments consists to overexpress one form of only one miRNA (at thousand copies). Thus, the definition of all the targets for a miRNA, called target site abundance (TSA) or the physiologic repertoire of RNAs targeted by a miRNA, depends on quantitative values for miRNA expression and should consider the percentage of ‘modified miRNA’. Moreover, the physiologic repertoire of RNAs targeted by a miRNA could change from a tissue to another [24].

To date, the in silico predicted RNA-targets of miRNAs are not all validated experimentally, suggesting that other critical events are involved in miRNA activity regulation or that some of the predictions are false positive. Among them, the post-transcriptional modifications of miRNAs add another level of fine-tuning gene expression.

The aim of this review is to focus on the other mechanisms favoring the miRNA diversity including post-transcriptional A-to-I editing mediated by the enzyme adenosine deaminase acting on RNA (ADAR), terminal trimming of nucleotides, or non-templated nucleotide additions (NTA). These mechanisms increase the repertoire of cellular regulatory miRNAs and provide additional plasticity to miRNA-mediated regulation of gene expression [15].

## 3. The Set of RNA Modifications among Bacterial Trans-Acting Small RNAs and Eukaryotic Micro RNAs

### 3.1. Internal RNA Mods

#### 3.1.1. sRNAs: Pseudouridine, Uracil to Thymidine (C5-Methylation, m^5^C)

Some regulatory RNAs ubiquitously present in bacteria, such as transfer messenger RNA (tmRNA), contain RNA modifications. In bacteria three ribosome rescue systems have been identified, including ubiquitous *trans*-translation system mediated by tmRNA and small protein B (SmpB). Ribosome rescue systems recycle ribosomes that stall at the 3′ end of truncated or non-stop mRNAs continuously arising in cells due to premature transcription termination or mRNA damage [25]. Two post-transcriptional modifications, 5-methyluridine and pseudouridine (φ), were detected in the tRNA-like portion of the RNA in *Escherichia coli* [26]. φ is referred to as the fifth ribonucleoside [27]. They enhance structural mimicry with native tRNA acceptor branches that all possess φ at similar locations, “rigidifying” the nearby sugar-phosphate backbone and enhancing base stacking in RNAs. Those two RNA modifications may facilitate alanylation by the alanyl-ARNt synthetase, recognition, and cargo to the stalled ribosomes by Elongation factor-Tu (EF-Tu) for ribosome rescue. They are probably added by tRNA-specific methyl transferase and pseudouridine synthase acting on canonical tRNAs, respectively. 5-methylcytidine (m^5^C) can change the coding sequence, alter splicing patterns or change RNA stability. m^5^C sites were detected within archaeal and bacterial mRNAs by bisulfite treatment of RNAs combined with high-throughput sequencing [28]. Investigations focusing on the putative presence of m^5^C within sRNAs should be performed. 

#### 3.1.2. Micro RNAs: A to I Editing, *N*^6^-methyladenosine

Most editing events in human occurs in UTRs. Adenosine-to-inosine (A-to-I) RNA editing also regulates miRNA biogenesis and function [29]. This epigenetic modification is performed by the adenosine deaminase acting on RNA (ADAR) enzymes and involved in the deamination of adenosine to inosine. These subtle miRNA post-transcriptional modifications concern only a minor fraction of the total pool of expressed miRNAs but those, once installed, provide a large array of regulatory outcomes than naked miRNAs [29]. 

In prokaryotes, much of the editing identified at present occur more predominantly in mRNAs and tRNAs. A-to-I RNA editing was reported to occur in tRNAs. Recent evidences identified RNA editing also in protein-coding genes in bacteria, especially one occurring in a toxic mRNA (*Hok*) regulated by an RNA antitoxin [30], as post-segregational killing mechanism employed by plasmids for their maintenance within the bacterial host cells. These newly discovered editing events that recode a Tyr into a Cys codon enhances toxicity of the encoded toxic peptides. Perhaps these recent findings can be envisioned as the tip of the iceberg and are plentiful in bacterial mRNA toxins to modulate toxicity of their expressed peptides, some secreted and acting as virulence factors during infection. Additionally, editing in mRNAs could influence the ability of these targets to be regulated by their regulatory sRNAs, influencing the strength of the pairing interactions. 

The resulting A-to-I conversions replace A-U Watson-Crick pairs with I•U wobble pairs in the double-strand RNA (dsRNA). Although I•U and isosteric G•U wobble base pairs, like Watson-Crick pairs, participate in forming helical regions in RNA folding, they seem to have unique conformational and biological features [31]. An inosine residue converted from adenosine in RNA is detected as an A-to-G change of the complementary DNA sequence, and the translation machinery reads inosine as guanosine, leading to alterations of codons.

ADARs can bind dsRNA regions of protein-coding genes and noncoding sequences, particularly pri-miRNAs [32]. Editing in pri-miRNA regulates the processing of miRNA [33,34]. Interestingly, the A-to-I editing alter mostly miRNAs that derive from an imprinted locus that includes 36 miRNA genes [35]. Editing of pri-miR-142 resulted in suppression of its processing by Drosha. The edited pri-miR-142 seems to be degraded by a component of RISC and a ribonuclease specific to inosine-containing dsRNAs [34]. In accordance with this observation, the expression level of mature miRNA-142 increases in ADAR1 null or ADAR2 null mice. It is also important to keep in mind that miRNA editing also seems to disturb asymmetric strand selection.

Editing in mature miRNA seems to lead to the selection of new target genes, modifying the initial repertoire for the miRNA [29,35]. The exact nature of edited miRNA is not well-established. A quantitative atlas of miRNA A-to-I editing has been recently published [36]. In this study, fewer than 160 miRNA editing sites have been identified. Authors identified 4162 sites present in ~80% of the pri-miRNAs and 574 sites in mature miRNAs. miRNA editing is prevalent in many tissue types in human. However, a high level of edited RNAs is found in neuronal tissues in mouse and *Drosophila*. This result agrees with the expression pattern of ADAR, which are preferentially expressed in neural tissues [37]. Authors showed that edited miRNAs in neuronal and non-neuronal tissues in humans gain two distinct sets of new targets, which are significantly associated with cognitive and organ developmental functions, respectively. Recently, another study systematically characterized miRNA editing in 10,593 human samples across 32 cancer types and normal controls [38]. Edited miRNAs tend to suppress expression of oncogenes. Moreover, authors observed a clear global tendency for hypo-editing in tumors allowing an accurate classification of normal/tumor samples based on their miRNA editing profile. Authors noted that thousands of miRNA binding sites are also differentially edited in cancer. 

**Figure 3:**
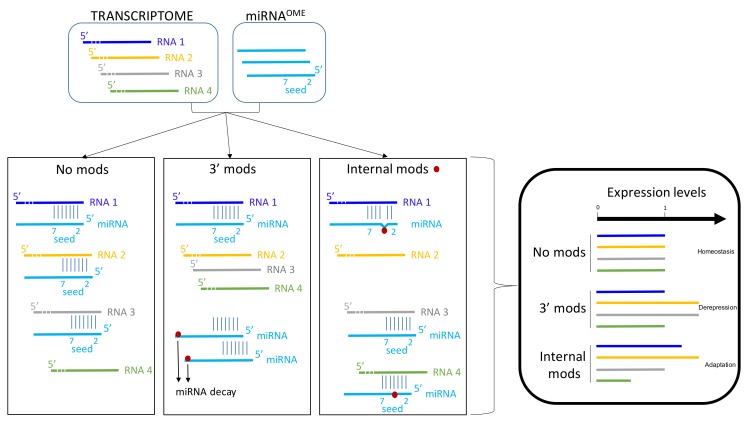
Epigenetic modifications and functional outcomes for miRNA and sRNA. Several epigenetic modifications might affect miRNA and sRNA functions. The majority of these RNAs are not modified. 3′ mods may promote miRNA decay. Internal mods, especially in the seed region of miRNA, may modify the pairing between miRNA and its targets (RNAs). In basal condition (homeostasis), a miRNA (here in blue) can bind to RNA 1–3. RNA 4 is not targeted by this miRNA. Expression levels of these four RNAs is arbitrarily estimated to 1 in basal condition. The decrease of miRNA amount per cell is associated with a loss or a decrease of miRNA effect on the targets. In other words, a transient upregulation (or de-repression) of the targets could occur in response to 3′ end modifications of miRNAs. This effect is supposed to be transient since usually miRNA are constitutively expressed. In contrast, internal modifications of miRNAs can modify the base-pairing with their targets. Consequently, several targets are regulated by these modified miRNAs and others are no more targeted. These internal modifications might promote the recognition of new targets. Altogether, these modifications alter the canonical repertoire of miRNAs. This type of modifications might favor the adaptation to the environment variations.

To resume, it seems that precursors of miRNA could be A-to-I edited on one or multiple sites. Consequently, the miRNA-processing machinery could be impaired or not. This post-transcriptional modification could annihilate the expression level of a miRNA, thus modifying the expression level of the targeted-RNAs by this miRNA (de-repression) [39]. If the A-to-I has no impact on miRNA production, the A-to-I editing of miRNA could modify the spectrum of RNA-targeted by this miRNA; especially if the seed sequence of the miRNA is affected (Figure 3). By this way, it could lead to de-repression of several RNAs and repression of other RNAs (new repertoire) [40]. Collectively, these data reinforce the role of RNA editing on gene regulation involving miRNAs. The A-to-I editing can provide an additional layer of complexity to the transcriptome, diversifying the genetic information beyond the linear sequence encoded in the genome.

#### 3.1.3. Adenosines (m6A) and Uridines (m^5^U) Methylations

##### 3.1.3.1. *N*^6^-methyladenosine

More than 160 types of chemical modifications in RNA are known. Recently, several modifications, such as *N*^6^-methyladenosine (m^6^A) [41], were detected in miRNA, highlighting the functional importance of epitranscriptomics [42]. The biological consequences of m^6^A are for the most part, unknown. However, m6A influences mRNA splicing, stability or translation [43]. What is the m6A cycle? m^6^A modification depends on three type of proteins; the ‘‘writer” (adenosine methyltransferase), ‘‘eraser” (m^6^A demethylating enzyme), and ‘‘reader” (m^6^A-binding protein) [42]. The m^6^A modification is reversibly installed and removed by writers and erasers, respectively. Even if the ‘m^6^A cycle’ is dynamic, the exact mechanisms regulating its timing are not elucidated.

##### 3.1.3.2. m6A Expression and Localization

The majority (~95%) of m^6^A peaks occurs within intragenic regions. m^6^A peaks are abundant in coding sequences (CDS; 50.9%), and untranslated regions (UTRs; 41.9%), with relatively few in intronic regions (2.0%) [44]. The m^6^A modification is commonly found within the conserved motif containing RRACH (A for methylated; R = purine, and H = A, C or U). Mapping of m^6^A in human and mouse RNAs has identified over 18,000 m^6^A sites in the transcripts of more than 7000 humans. 

Predominant consensus motifs of m^6^A: G [G/A] m6ACU and related variants ([AC]GAC[GU], GGAC, [AU][CG]G[AG]AC and UGAC), and almost 90% of all m^6^A peaks contain at least one of the motifs [45]. The localization of individual m6A sites in many mRNAs is highly similar between human and mouse, and transcriptome-wide analysis reveals that m^6^A is found in regions of high evolutionary conservation. m^6^A is found within long internal exons and is preferentially enriched within 3′ -UTRs and around stop codons.

##### 3.1.3.3. m^6^A Writers

The methyltransferase-like 3 (METTL3) plays a critical role in m^6^A writing [46]. However, this enzyme requires at least the METTL14, a pseudo-methyl transferase protein to form a stable complex to efficiently deposit m^6^A on RNAs. Even if the knock-down of these two proteins strongly affect the m^6^A abundance in mammalian cells, other proteins belongs to the ‘m^6^A writer complex’ such as RBM15 and WTAP proteins [43]. The exact function of these proteins is poorly understood, and the complex composition may fluctuate in function of RNA environment.

##### 3.1.3.4. m^6^A Readers

Several proteins termed Readers have been described to recognize the m^6^A motif [47]. These proteins belong to the YT521-B homology (YTH) family, which selectively bind to m^6^A-modified RNAs and affect their functions. In mammals, there are five members in the YTH family, i.e., YTHDC1, YTHDC2, YTHDF1, YTHDF2, and YTHDF3. Interestingly, YTHDC1 is localized in the nucleus in contrast to the other in the cytoplasm. YTHDF1, YTHDF3, and YTHDC2 selectively promote the translation efficiency of m^6^A-containing mRNAs. In contrast, YTHDF2 decreases the mRNA stability by favoring deadenylations. Insulin-like growth factor-2 mRNA-binding proteins 1, 2, and 3 (IGF2BP1–3) also behave as m^6^A readers. IGF2BPs selectively recognize m^6^A-containing RNAs and promote their translation and stability [48].

##### 3.1.3.5. m^6^A Erasers

AlkB homolog 5 (ALKBH5) [49] and the fat mass and obesity associated protein (FTO) [50] erase m^6^A on RNA (here considered as a demethylation). Even if these ALKBH5 and FTO erase the m^6^A motif on RNA, the FTO requires two additional intermediates to obtain an adenosine residue from a m^6^A. Importantly, FTO is involved in other RNA demethylation processes (3-methyluracil (3-meU) and N6,2′-O-dimethyladenosine (m^6^Am)) [51].

##### 3.1.3.6. m^6^A and Micro RNAs

m^6^A is crucial for miRNA biogenesis and expression levels [52]. Pri-miRNAs have an over-representation of GGAC motif in their sequences. This motif is consistent with the established recognition sequence RGAC for RNA methyltransferase METTL3 (methyltransferase-like 3). METTL3 methylates pri-miRNAs, marking them for recognition and processing by RNA binding protein DGCR8. Microprocessor complex, made of the DGCR8 and the ribonuclease type III DROSHA, binds to methylated pri-miRNAs to yield the pre-miRNAs. METTL3 depletion reduced the binding of DGCR8 to pri-miRNAs and resulted in the global reduction of mature miRNAs and concomitant accumulation of unprocessed pri-miRNAs. Conversely, gain-of-function experiments revealed that METTL3 is enough to enhance miRNA maturation. m^6^A-dependant production of miRNA is global and non-cell-type specific, suggesting that m^6^A provides additional global functions to the miRNAs.

##### 3.1.3.7. Consequences of m^6^A on Micro RNA Activity

Since miRNAs target mRNAs by binding at their 3′-UTRs in a complex with Ago proteins, this suggests a potential role of mRNA methylation in the miRNA-mediated expression inhibition of mRNA targets.

It is noteworthy that two-third of the mRNAs which contain a m^6^A site within their 3′-UTR also have at least one miRNA binding site in their 3′-UTRs [45]. Intriguingly, it has been described that in 3′-UTRs with both m^6^A peaks and miRNA binding sites, the m^6^A peaks precede miRNA binding sites ~60% of the time. m^6^A peaks in mRNAs are abundant near the stop codon and generally decrease in frequency along 3′-UTRs. In the other hand, miRNA targeting miRNA binding sites located in the first hundred nucleotides in 3′-UTR (near the stop codon) seem to have a stronger effect than the other. Thus, it is tempting to speculate that m^6^A may influence the function or the binding of miRNAs in 3′-UTR. By this way, m^6^A could promote the mRNA stability at least by limiting the miRNA binding on these 3′-UTR. In accordance with this idea, m^6^A has been found enriched in ribosome-associated mRNA [53].

m^6^A is also detected in bacterial ribosomal RNAs, mRNAs and some sRNAs [54]. Transcriptome-wide m^6^A profiling in *E. coli* and *Pseudomonas aeruginosa* revealed a conserved m^6^A pattern that is distinct from those in eukaryotes, with a unique GCCAU sequence consensus motif. RsmY and RsmZ sRNAs are enriched in the *P. aeruginosa* ‘methylome’. These two sRNAs sequester RNA-binding proteins and are central regulatory elements in signal transduction pathways controlling virulence and the selection of favorable carbon sources [55]. m^6^A RNA methylation of RsmY and RsmZ RNAs could modulate their binding affinity with their protein ligands.

In recent years, a variety of methyltransferases was identified in bacteria, which play important roles in various functional pathways. Some enzymes, as RlmF and RlmJ, methylate selected adenines in 16S [56] and 23S [57] rRNAs from *E. coli*. Some methyltransferases are conserved in many species of eubacteria, eukaryotes, and archaea [56]. As an example, KsgA is an rRNA post-transcriptional modification enzyme that catalyzes the transfer of four methyl groups from S-adenosyl-l-methionine to two adjacent adenosine bases in 16S rRNA. KsgA seems conserved in all three domains of life, a conservation that extends further into mitochondria and chloroplasts.

#### 3.1.4. 3′-Terminal Untemplated Nucleotide Addition

RNAs undergo multiple types of tailing, including adenylation, uridylation, and guanylation [58]. Pioneering studies on the 3′-terminome of RNA have revealed new layers of gene expression [59].

### 3.2. Mods at RNA Ends

#### 3.2.1. sRNAs: 5′-NAD-capped RNA

Whereas miRNAs do not carry specific RNA modifications at their 5′-ends, some sRNAs do. Although many eukaryotic transcripts contain cap structures, it has been long thought that bacterial RNAs do not carry any special modifications on their 5′-ends. In bacteria, primary transcripts are produced by transcription initiated with a nucleoside triphosphate and are triphosphorylated on 5′-ends. In addition to mono- and triphosphorylated moieties, some mRNAs and sRNAs contain cap-like structures and diphosphates on their 5′-ends. Bacterial RNA polymerase can initiate transcription with adenosine-containing cofactor NAD^+^ (nicotinamide adenine dinucleotide), resulting in a portion of cellular RNAs (up to 25% in specific RNAs) being ‘capped’ at their 5′ ends with NAD^+^ [60,61], reminiscent of the 7-methylguanylate eukaryotic cap. 5′-CoA can also occur instead of the characteristic 5′-triphosphate or 5′-monophosphate groups [62]. 5′-NAD-capped RNAs are detected in sRNAs and mRNAs, but not in rRNAs or tRNAs. The NAD cap is incorporated into RNA transcripts by the RNA polymerase and later removed by the Nudix family hydrolase NudC. The physiological significance of these modifications, however, remains unclear.

#### 3.2.2. Micro RNAs: 3′-Uridinylation

RNA uridylation is a widespread posttranscriptional regulator of gene expression in eukaryotes [63] but is not reported in prokaryotic sRNAs. RNA uridylation is catalyzed by the terminal uridylyltransferases (TUTases) by adding uridines at the 3′ end of target transcripts. TUTases belong to the DNA polymerase β (Pol β)-like nucleotidyltransferase superfamily, which include RNA-specific nucleotidyltransferases divided in three subgroups. The TUTases, members of subgroup 2, can recognize a diversity of RNA substrates, such as mitochondria and noncoding and mRNAs. RNA uridylation were detected in a range of eukaryotes, including, animals, plants, and fungi [64]. Virtually all classes of eukaryotic RNAs can be uridylated. The addition of a few uridines at the 3′ end of an RNA influences its stability. The usual consequence of uridylation on RNAs or their precursors is to accelerate decay by facilitating the recruitment of exonucleases via U tails [65]. Even if the role of uridylation on mRNA is clearly established, the impact of uridylation on pri-miRNA or miRNA is less studied.

##### 3.2.2.1. Micro RNA and Mono-Uridylation

Monouridylation can occur during miRNA biogenesis. Unlike prototypic pre-miRNAs (group 1), group 2 pre-miRNAs acquire a shorter (1nt) 3′ overhang from Drosha processing and therefore require a 3′ end mono-uridylation, for Dicer processing [12]. TUT7/ZCCHC6, TUT4/ZCCHC11, and TUT2/PAPD4/GLD2 as the terminal uridylyl transferases are responsible for pre-miRNA mono-uridylation. The TUTs act specifically on dsRNAs with a 1nt 3′ overhang, thereby creating a 2 nt 3′ overhang.

This mono-uridylation seems to be specific to some miRNAs. The majority of let-7 and miR-105 miRNA per cell seems to be mono-uridylated [66]. Mono-uridylation could protect the pre-miRNA (1nt 3′ overhang) generated by Drosha from 3′-exonuclease-mediated trimming and enhances Dicer processing. However, the exact number of pre-miRNAs needing mono-uridylation on 3′ overhang is still unknown. In addition, TUT2 have been described to mono-adenylate and stabilize mature miR-122 in mammalian liver cells and fibroblasts [67,68], suggesting that this 1nt addition is not restricted to rare pre-miRNAs or low-expressed miRNAs. 

However, in vivo gene knockout studies coupled with deep sequencing have revealed that the TUT ZCCHC11 uridylates the 3′ ends of many mature miRNAs [69]. The addition of a single uridine to miR-126-5p or miR-379 reduced their silencing activity against insulin-like growth factor-1 (IGF-1) mRNAs and had a cumulative effect when multiple uridylated miRNAs targeted a single transcript [69]. Similar results have been recently published in human lymphocytes [70]. TUT4 is essential to catalyze 3′ non-templated nucleotide addition (3′NTA) to miRNAs in the steady state of T lymphocytes. Upon T-cell activation, miRNA sequences bearing terminal uridines are specifically decreased, concomitantly with down-regulation of TUT4 and TUT7 enzymes. Convincingly, analysis of synthetic uridylated miRNAs shows that 3′ addition of uridines promotes degradation of these uridylated miRNAs after T-cell activation.

Altogether, these data underline post-transcriptional uridylation as a mechanism to fine-tune miRNA levels.

##### 3.2.2.2. Pre-Micro RNA and Oligo-Uridylation

Several miRNAs are mono-uridylated but TUTases also target pre-miRNA. let-7 maturation is inhibited by an RNA-binding protein Lin28 [66,71]. Lin28A (one human homolog of lin28) is expressed in embryonic cells and cancer cells. Lin28A binds to the terminal loop of let-7 precursors through a conserved motif GGAG to interfere with Drosha and Dicer processing [72]. Lin28 also interacts with TUT4 to induce oligo-uridylation (10–30 nt) of pre-let-7 [73]. Since oligo-uridylated miRNA are less processed by Dicer, the oligo(U) tail serves as a decay signal for the Perlman syndrome exonuclease DIS3L2 in order to suppress let-7 maturation [74].

#### 3.2.3. sRNA Stabilization by 3′ Polyadenylation in Prokaryotes

The transient addition of poly(A) tails to bacterial RNAs is essential for the 3′ exonucleolytic degradation of stem-loop structures in decay intermediates [75]. This holds true for the 3′ adenylation of antisense RNAs that regulate plasmid replication in *E. coli* that greatly accelerates their decay [76]. More generally, recent evidences suggest that poly(A) polymerase which promotes RNA degradation by exoribonucleases through the addition of poly(A) tails, has essential roles in the regulation of gene expression by Hfq-dependent sRNAs in *E. coli* [77]. Poly(A) polymerase stabilizes some sRNAs in *E. coli* by blocking their RNase E-mediated decays, but it is currently unknown if this observation can be extended to other bacterial sRNAs, especially those expressed by Gram-positive bacteria. 

## 4. Concluding Remarks and Outlook

sRNA and miRNA are thought to be involved in fine-tuning of gene expression. In eukaryotes, loss of function of miRNA genes is usually not associated to phenotypes, as for prokaryotic sRNAs. Less than 10% of knockout mice for evolutionarily-conserved miRNAs induce developmental or abnormal embryonic phenotypes [78], although they probably will be detected in sensitized backgrounds or under stress. Similar findings have been reported in *Caenorhabditis elegans* [79,80] and in plants [81]. Added to the generally weak post-transcriptional effect of miRNAs on their targets (5%–30%) [20], these results led to the presentation of miRNAs as cellular actors responsible for fine tuning of gene expression levels. However, it is also possible that compensations occur in knock out experiments since seed sequences are short and more than a thousand miRNAs are currently known in humans [16,82]. In the case of sRNAs, similar conclusions can be drawn concerning their fine-tuning roles on gene expression, the lack of strong associated phenotypes, but current estimations of their amount per bacterial cell are in the hundreds, tenfold less than miRNAs, but acting on ~1000-fold smaller genomes. Each sRNA and miRNA possess multiple targets, and the expression of a given RNA target can be regulated by multiple sRNAs or miRNAs.

Nevertheless, co-evolution of coding and miRNA genes have been described in almost all models studied, suggesting that small RNAs provide advantages for fitness and adaptation [83]. Since it is rare than a miRNA affected more than ~30% of its target RNA expression levels, therefore mainly implicated in fine-tuning of gene expression in response to environmental triggers. Thus, these sRNAs are ‘transcriptomic adjusters’, in accordance with their expression levels. They are already present in the cell before the environmental stress occurs, suggesting an immediate and reversible effect on their targets. The basal expression level is usually low for miRNAs and sRNAs (50–500 copies/cell) but their expression level can be significantly modulated (induction or repression) via transcription or epigenetic modifications, including RNA modifications [4].

Epigenetic modifications on small RNAs can generate three outputs: (1) reduction of sRNA amounts by inhibiting their biogenesis or increasing their turnovers, (2) increase their stability, or (3) sequence modifications enhancing or reducing pairing with their canonical or alternative targets. Thus, epigenetics events induced by stresses will alter the magnitude of effects and repertoire of small RNAs expressed (Figure 3). Reciprocally, epigenetic modifications of RNAs alter miRNA or sRNA binding to their respective targets.

Nevertheless, the exact nature and number of small RNAs affected by epigenetic modifications is not yet established accurately, despite massive RNA sequencing experiments. Only subsets of miRNAs [84] and sRNAs are modified. The proportion and nature of epigenetic modifications for miRNAs are linked to the tissues that express them, the organism and the environmental stress implicated. Moreover, for a miRNA, only a fraction is altered [85], probably as for sRNAs in bacteria. In addition, different epigenetic modifications can be removed concomitantly for a given miRNA [86]. These results advocate that it is difficult to establish the full target repertoire for a microRNA. To resume, the combination of many different miRNAs or sRNAs and the diversity of epigenetic modifications for miRNAs offer an elegant and efficient manner to monitor and fine-tune gene expression. Regulators and targeted RNAs are both exposed to epigenetic modifications, which substantially increase the combinatorial possibilities and outcomes, especially if the modifications affect the pairing between the sRNA and its target.

Important questions remain unsolved: the delay between miRNA-target hybridization and mRNA decay [87]. To date, it is difficult to establish the timing between miRNA pairing, translation inhibition, and/or mRNA decay. Since transcription and translation are coupled in bacteria, but disconnected in eukaryotes due to cell compartmentalization, that delay should be shorter for bacterial sRNA than for miRNAs. Many players are required for such mi/sRNA gene regulations. Moreover, some epigenetic modifications can be reverted [88], suggesting that epigenetic modifications could be cheaper energy wise than neo-transcription. In addition, these modifications are inserted faster than neo-transcription, which is an advantage to respond quickly to an environmental stress (Figure 3). 

Collectively, these epigenetics modifications provide survival advantages. Curiously, epigenetic modifications can differ when models are compared [84]. Even if we provide striking examples of similarity between sRNA and miRNA biogenesis, mechanisms, functions, and decays, there are also differences in term of their biogenesis and actions. A recent study attempted to identify proteins involved in miRNA biogenesis in pre-metazoan model [89]. They demonstrated that the microprocessor complex evolved long before the last common ancestor of animals, consistent with a pre-metazoan origin of most of the animal developmental gene elements. Through small RNA sequencing, they discovered expressed bona fide miRNA genes in several species of the ichthyosporeans (unicellular osmotrophic feeders).

In bacteria, the existence of miRNA analogues, beside sRNAs, is poorly studied. Small RNAs (15–28 nt long), considered as RNA fragments, were identified in some bacteria [90], but whether they are considered as transcriptional noise or bona fide sRNA is unclear for now. A phage-derived microRNA-size (20 nt long RNA), was isolated from an *E. coli* culture after induction of Shiga toxin-converting bacteriophage Φ24B [91]. Phages from this group are involved in virulence of *E. coli* enterohemorrhagic strains, giving physiological significance of a microRNA-size molecule in bacterial cells. Whether this recent example corresponds to the tip of the iceberg or an exception is unknown. 

## References

[1] Prasanth K.V., Spector D.L. (2007). Eukaryotic regulatory RNAs: an answer to the ’genome complexity’ conundrum. Genes Dev..

[2] Gelsinger D.R., DiRuggiero J. (2018). Transcriptional Landscape and Regulatory Roles of Small Noncoding RNAs in the Oxidative Stress Response of the Haloarchaeon *Haloferax volcanii*.. J Bacteriol..

[3] Waters L.S., Storz G. (2009). Regulatory RNAs in bacteria. Cell.

[4] Bartel D.P. (2018). Metazoan microRNAs. Cell.

[5] Georg J., Hess W.R. (2011). *cis*-antisense RNA, another level of gene regulation in bacteria. Microbiol Mol Biol Rev..

[6] Helm M., Motorin Y. (2017). Detecting RNA modifications in the epitranscriptome: predict and validate. Nat Rev Genet..

[7] Wagner E.G.H., Romby P. (2015). Small RNAs in bacteria and archaea. Advances in Genetics.

[8] Zhao X., Zhang Y., Huang X. (2018). Pathogenicity-island-encoded regulatory RNAs regulate bacterial virulence and pathogenesis. Microb. Pathog..

[9] Felden B., Cattoir V. (2018). Bacterial adaptation to antibiotics through regulatory RNAs. Antimicrob. Agents Chemother..

[10] Carrier M.-C., Lalaouna D., Massé E. (2018). Broadening the definition of bacterial small RNAs: Characteristics and mechanisms of action. Annu. Rev. Microbiol..

[11] Santiago-Frangos A., Woodson S.A. (2018). Hfq chaperone brings speed dating to bacterial sRNA. Wiley interdisciplinary reviews. RNA.

[12] Kim Y.-K., Kim B., Kim V.N. (2016). Re-evaluation of the roles of DROSHA, Exportin 5, and DICER in microRNA biogenesis. Proc. Natl. Acad. Sci. USA.

[13] Winter J., Jung S., Keller S., Gregory R.I., Diederichs S. (2009). Many roads to maturity: microRNA biogenesis pathways and their regulation. Nat. Cell Biol..

[14] Wang Y., Juranek S., Li H., Sheng G., Wardle G.S., Tuschl T., Patel D.J. (2009). Nucleation, propagation and cleavage of target RNAs in Ago silencing complexes. Nature.

[15] Ameres S.L., Zamore P.D. (2013). Diversifying microRNA sequence and function. Nature reviews. Mol. Cell Biol..

[16] Agarwal V., Bell G.W., Nam J.-W., Bartel D.P. (2015). Predicting effective microRNA target sites in mammalian mRNAs. eLife.

[17] Seok H., Ham J., Jang E.-S., Chi S.W. (2016). MicroRNA target recognition: Insights from transcriptome-wide non-canonical interactions. Mol. Cells.

[18] Helwak A., Kudla G., Dudnakova T., Tollervey D. (2013). Mapping the human miRNA interactome by CLASH reveals frequent noncanonical binding. Cell.

[19] Khorshid M., Hausser J., Zavolan M., van Nimwegen E. (2013). A biophysical miRNA-mRNA interaction model infers canonical and noncanonical targets. Nat. Methods.

[20] Loeb G.B., Khan A.A., Canner D., Hiatt J.B., Shendure J., Darnell R.B., Leslie C.S., Rudensky A.Y. (2012). Transcriptome-wide miR-155 binding map reveals widespread noncanonical microRNA targeting. Mol. Cell.

[21] Kim D., Sung Y.M., Park J., Kim S., Kim J., Park J., Ha H., Bae J.Y., Kim S., Baek D. (2016). General rules for functional microRNA targeting. Nat. Genet..

[22] Salmena L., Poliseno L., Tay Y., Kats L., Pandolfi P.P. (2011). A ceRNA hypothesis: The Rosetta Stone of a hidden RNA language?. Cell.

[23] Tay Y., Rinn J., Pandolfi P.P. (2014). The multilayered complexity of ceRNA crosstalk and competition. Nature.

[24] Chi S.W., Zang J.B., Mele A., Darnell R.B. (2009). Argonaute HITS-CLIP decodes microRNA-mRNA interaction maps. Nature.

[25] Huter P., Müller C., Arenz S., Beckert B., Wilson D.N. (2017). Structural basis for ribosome rescue in bacteria. Trends Biochem. Sci..

[26] Felden B., Hanawa K., Atkins J.F., Himeno H., Muto A., Gesteland R.F., McCloskey J.A., Crain P.F. (1998). Presence and location of modified nucleotides in *Escherichia coli* tmRNA: Structural mimicry with tRNA acceptor branches. EMBO J..

[27] Cohn W.E. (1960). Pseudouridine, a carbon-carbon linked ribonucleoside in ribonucleic acids: Isolation, structure, and chemical characteristics. J. Biol. Chem..

[28] Edelheit S., Schwartz S., Mumbach M.R., Wurtzel O., Sorek R. (2013). Transcriptome-wide mapping of 5-methylcytidine RNA modifications in bacteria, archaea, and yeast reveals m^5^C within archaeal mRNAs. PLoS Genet..

[29] Wulff B.-E., Nishikura K. (2011). Modulation of MicroRNA expression and function by ADARs. Current Topics in Microbiology and Immunology.

[30] Bar-Yaacov D., Mordret E., Towers R., Biniashvili T., Soyris C., Schwartz S., Dahan O., Pilpel Y. (2017). RNA editing in bacteria recodes multiple proteins and regulates an evolutionarily conserved toxin-antitoxin system. Genome Res..

[31] Ananth P., Goldsmith G., Yathindra N. (2013). An innate twist between Crick’s wobble and Watson-Crick base pairs. RNA.

[32] Nishikura K. (2016). A-to-I editing of coding and non-coding RNAs by ADARs. Nat. Rev. Mol. Cell Biol..

[33] Kong W., Yang H., He L., Zhao J., Coppola D., Dalton W.S., Cheng J.Q. (2008). MicroRNA-155 is regulated by the transforming growth factor β/Smad pathway and contributes to epithelial cell plasticity by targeting RhoA. Mol. Cell. Biol..

[34] Yang W., Chendrimada T.P., Wang Q., Higuchi M., Seeburg P.H., Shiekhattar R., Nishikura K. (2006). Modulation of microRNA processing and expression through RNA editing by ADAR deaminases. Nat. Struct. Mol. Biol..

[35] Kawahara Y., Zinshteyn B., Sethupathy P., Iizasa H., Hatzigeorgiou A.G., Nishikura K. (2007). Redirection of silencing targets by adenosine-to-inosine editing of miRNAs. Science.

[36] Li L., Song Y., Shi X., Liu J., Xiong S., Chen W., Fu Q., Huang Z., Gu N., Zhang R. (2018). The landscape of miRNA editing in animals and its impact on miRNA biogenesis and targeting. Genome Res..

[37] Goldstein B., Agranat-Tamir L., Light D., Ben-Naim Zgayer O., Fishman A., Lamm A.T. (2017). A-to-I RNA editing promotes developmental stage–specific gene and lncRNA expression. Genome Res..

[38] Pinto Y., Buchumenski I., Levanon E.Y., Eisenberg E. (2018). Human cancer tissues exhibit reduced A-to-I editing of miRNAs coupled with elevated editing of their targets. Nucleic Acids Res..

[39] Zhang L., Yang C.-S., Varelas X., Monti S. (2016). Altered RNA editing in 3′ UTR perturbs microRNA-mediated regulation of oncogenes and tumor-suppressors. Sci. Rep..

[40] Brümmer A., Yang Y., Chan T.W., Xiao X. (2017). Structure-mediated modulation of mRNA abundance by A-to-I editing. Nat. Commun..

[41] Desrosiers R., Friderici K., Rottman F. (1974). Identification of methylated nucleosides in messenger RNA from Novikoff hepatoma cells. Proc. Natl. Acad. Sci. USA.

[42] Roignant J.-Y., Soller M. (2017). m^6^A in mRNA: An ancient mechanism for fine-tuning gene expression. Trends Genet..

[43] Huang J., Yin P. (2018). Structural insights into *N*^6^-methyladenosine (m^6^A) modification in the transcriptome. Genom. Proteom. Bioinform..

[44] Dominissini D., Moshitch-Moshkovitz S., Schwartz S., Salmon-Divon M., Ungar L., Osenberg S., Cesarkas K., Jacob-Hirsch J., Amariglio N., Kupiec M. (2012). Topology of the human and mouse m^6^A RNA methylomes revealed by m^6^A-seq. Nature.

[45] Meyer K.D., Saletore Y., Zumbo P., Elemento O., Mason C.E., Jaffrey S.R. (2012). Comprehensive analysis of mRNA methylation reveals enrichment in 3′ UTRs and near stop codons. Cell.

[46] Bokar J.A., Rath-Shambaugh M.E., Ludwiczak R., Narayan P., Rottman F. (1994). Characterization and partial purification of mRNA N6-adenosine methyltransferase from HeLa cell nuclei. Internal mRNA methylation requires a multisubunit complex. J. Biol. Chem..

[47] Edupuganti R.R., Geiger S., Lindeboom R.G.H., Shi H., Hsu P.J., Lu Z., Wang S.-Y., Baltissen M.P.A., Jansen P.W.T.C., Rossa M. (2017). *N*^6^-methyladenosine (m^6^A) recruits and repels proteins to regulate mRNA homeostasis. Nat. Struct. Mol. Biol..

[48] Huang H., Weng H., Sun W., Qin X., Shi H., Wu H., Zhao B.S., Mesquita A., Liu C., Yuan C.L. (2018). Recognition of RNA *N*^6^-methyladenosine by IGF2BP proteins enhances mRNA stability and translation. Nat. Cell Biol..

[49] Zheng G., Dahl J.A., Niu Y., Fedorcsak P., Huang C.-M., Li C.J., Vågbø C.B., Shi Y., Wang W.-L., Song S.-H. (2013). ALKBH5 is a mammalian RNA demethylase that impacts RNA metabolism and mouse fertility. Mol. Cell.

[50] Jia G., Fu Y., Zhao X., Dai Q., Zheng G., Yang Y., Yi C., Lindahl T., Pan T., Yang Y.-G. (2011). N6-methyladenosine in nuclear RNA is a major substrate of the obesity-associated FTO. Nat. Chem. Biol..

[51] Wei J., Liu F., Lu Z., Fei Q., Ai Y., He P.C., Shi H., Cui X., Su R., Klungland A. (2018). Differential m^6^A, m^6^A_m_, and m^1^A demethylation mediated by FTO in the cell nucleus and cytoplasm. Mol. Cell.

[52] Alarcón C.R., Lee H., Goodarzi H., Halberg N., Tavazoie S.F. (2015). *N*^6^-methyladenosine marks primary microRNAs for processing. Nature.

[53] Wang X., Zhao B.S., Roundtree I.A., Lu Z., Han D., Ma H., Weng X., Chen K., Shi H., He C. (2015). N^6^-methyladenosine modulates messenger RNA translation efficiency. Cell.

[54] Deng X., Chen K., Luo G.-Z., Weng X., Ji Q., Zhou T., He C. (2015). Widespread occurrence of *N*^6^-methyladenosine in bacterial mRNA. Nucleic Acids Res..

[55] Sonnleitner E., Haas D. (2011). Small RNAs as regulators of primary and secondary metabolism in *Pseudomonas* species. Appl. Microbiol. Biotechnol..

[56] O’Farrell H.C., Scarsdale J.N., Rife J.P. (2004). Crystal structure of KsgA, a universally conserved rRNA adenine dimethyltransferase in *Escherichia coli*.. J. Mol. Biol..

[57] Sergiev P.V., Serebryakova M.V., Bogdanov A.A., Dontsova O.A. (2008). The *ybiN* gene of *Escherichia coli* encodes adenine-N^6^ methyltransferase specific for modification of A1618 of 23 S ribosomal RNA, a methylated residue located close to the ribosomal exit tunnel. J. Mol. Biol..

[58] Chang H., Lim J., Ha M., Kim V.N. (2014). TAIL-seq: Genome-wide determination of poly(A) tail length and 3′ end modifications. Mol. Cell.

[59] Lee M., Kim B., Kim V.N. (2014). Emerging roles of RNA modification: M^6^A and U-tail. Cell.

[60] Cahová H., Winz M.-L., Höfer K., Nübel G., Jäschke A. (2015). NAD captureSeq indicates NAD as a bacterial cap for a subset of regulatory RNAs. Nature.

[61] Chen Y.G., Kowtoniuk W.E., Agarwal I., Shen Y., Liu D.R. (2009). LC/MS analysis of cellular RNA reveals NAD-linked RNA. Nat. Chem. Biol..

[62] Jäschke A., Höfer K., Nübel G., Frindert J. (2016). Cap-like structures in bacterial RNA and epitranscriptomic modification. Curr. Opin. Microbiol..

[63] De Almeida C., Scheer H., Zuber H., Gagliardi D. (2018). RNA uridylation: A key posttranscriptional modification shaping the coding and noncoding transcriptome. Wiley interdisciplinary reviews. RNA.

[64] Menezes M.R., Balzeau J., Hagan J.P. (2018). 3′ RNA uridylation in epitranscriptomics, gene regulation, and disease. Front. Mol. Biosci..

[65] Ji L., Chen X. (2012). Regulation of small RNA stability: Methylation and beyond. Cell Res..

[66] Heo I., Ha M., Lim J., Yoon M.-J., Park J.-E., Kwon S.C., Chang H., Kim V.N. (2012). Mono-uridylation of pre-microRNA as a key step in the biogenesis of group II let-7 microRNAs. Cell.

[67] Burns D.M., D’Ambrogio A., Nottrott S., Richter J.D. (2011). CPEB and two poly(A) polymerases control miR-122 stability and p53 mRNA translation. Nature.

[68] Katoh T., Sakaguchi Y., Miyauchi K., Suzuki T., Kashiwabara S.-I., Baba T., Suzuki T. (2009). Selective stabilization of mammalian microRNAs by 3′ adenylation mediated by the cytoplasmic poly(A) polymerase GLD-2. Genes Dev..

[69] Jones M.R., Blahna M.T., Kozlowski E., Matsuura K.Y., Ferrari J.D., Morris S.A., Powers J.T., Daley G.Q., Quinton L.J., Mizgerd J.P. (2012). Zcchc11 uridylates mature miRNAs to enhance neonatal IGF-1 expression, growth, and survival. PLoS Genet..

[70] Gutiérrez-Vázquez C., Enright A.J., Rodríguez-Galán A., Pérez-García A., Collier P., Jones M.R., Benes V., Mizgerd J.P., Mittelbrunn M., Ramiro A.R. (2017). 3′ Uridylation controls mature microRNA turnover during CD4 T-cell activation. RNA.

[71] Newman M.A., Thomson J.M., Hammond S.M. (2008). Lin-28 interaction with the Let-7 precursor loop mediates regulated microRNA processing. RNA.

[72] Loughlin F.E., Gebert L.F.R., Towbin H., Brunschweiger A., Hall J., Allain F.H.-T. (2011). Structural basis of pre-let-7 miRNA recognition by the zinc knuckles of pluripotency factor Lin28. Nat. Struct. Mol. Biol..

[73] Hagan J.P., Piskounova E., Gregory R.I. (2009). Lin28 recruits the TUTase Zcchc11 to inhibit let-7 maturation in mouse embryonic stem cells. Nat. Struct. Mol. Biol..

[74] Chang H.-M., Triboulet R., Thornton J.E., Gregory R.I. (2013). A role for the Perlman syndrome exonuclease Dis3l2 in the Lin28–let-7 pathway. Nature.

[75] Sarkar N. (1997). Polyadenylation of mRNA in prokaryotes. Annu. Rev. Biochem..

[76] Xu F., Cohen S.N. (1995). RNA degradation in *Escherichia coli* regulated by 3′ adenylation and 5′ phosphorylation. Nature.

[77] Sinha D., Matz L.M., Cameron T.A., De Lay N.R. (2018). Poly(A) polymerase is required for RyhB sRNA stability and function in *Escherichia coli*.. RNA.

[78] Park C.Y., Jeker L.T., Carver-Moore K., Oh A., Liu H.J., Cameron R., Richards H., Li Z., Adler D., Yoshinaga Y. (2012). A resource for the conditional ablation of microRNAs in the mouse. Cell Rep..

[79] Alvarez-Saavedra E., Horvitz H.R. (2010). Many families of *C. elegans* microRNAs are not essential for development or viability. Curr. Biol..

[80] Miska E.A., Alvarez-Saavedra E., Abbott A.L., Lau N.C., Hellman A.B., McGonagle S.M., Bartel D.P., Ambros V.R., Horvitz H.R. (2007). Most *Caenorhabditis elegans* microRNAs are individually not essential for development or viability. PLoS Genet..

[81] Reichel M., Li Y., Li J., Millar A.A. (2015). Inhibiting plant microRNA activity: Molecular SPONGEs, target MIMICs and STTMs all display variable efficacies against target microRNAs. Plant Biotechnol. J..

[82] Kozomara A., Griffiths-Jones S. (2014). MiRBase: Annotating high confidence microRNAs using deep sequencing data. Nucleic Acids Res..

[83] Li J., Liu Y., Xin X., Kim T.S., Cabeza E.A., Ren J., Nielsen R., Wrana J.L., Zhang Z. (2012). Evidence for positive selection on a number of microRNA regulatory interactions during recent human evolution. PLoS Genet..

[84] Sun W.-J., Li J.-H., Liu S., Wu J., Zhou H., Qu L.-H., Yang J.-H. (2016). RMBase: A resource for decoding the landscape of RNA modifications from high-throughput sequencing data. Nucleic Acids Res..

[85] Kleaveland B., Shi C.Y., Stefano J., Bartel D.P. (2018). A network of noncoding regulatory RNAs acts in the mammalian brain. Cell.

[86] Yates L.A., Norbury C.J., Gilbert R.J.C. (2013). The long and short of microRNA. Cell.

[87] Baek D., Villén J., Shin C., Camargo F.D., Gygi S.P., Bartel D.P. (2008). The impact of microRNAs on protein output. Nature.

[88] Höfer K., Jäschke A. (2018). Epitranscriptomics: RNA modifications in bacteria and archaea. Microbiol. Spectr..

[89] Bråte J., Neumann R.S., Fromm B., Haraldsen A.A.B., Tarver J.E., Suga H., Donoghue P.C.J., Peterson K.J., Ruiz-Trillo I., Grini P.E. (2018). Unicellular origin of the animal microRNA machinery. Curr. Biol..

[90] Kang S.-M., Choi J.-W., Lee Y., Hong S.-H., Lee H.-J. (2013). Identification of microRNA-size, small RNAs in *Escherichia coli*.. Curr. Microbiol..

[91] Nejman-Faleńczyk B., Bloch S., Licznerska K., Dydecka A., Felczykowska A., Topka G., Węgrzyn A., Węgrzyn G. (2015). A small, microRNA-size, ribonucleic acid regulating gene expression and development of Shiga toxin-converting bacteriophage Φ24Β. Sci. Rep..

